# A Single Basis for Developmental Buffering of *Drosophila* Wing Shape

**DOI:** 10.1371/journal.pone.0000007

**Published:** 2006-12-20

**Authors:** Casper J Breuker, James S Patterson, Christian Peter Klingenberg

**Affiliations:** Faculty of Life Sciences, University of Manchester Manchester, United Kingdom; Fred Hutchinson Cancer Research Center, United States of America

## Abstract

The nature of developmental buffering processes has been debated extensively, based on both theoretical reasoning and empirical studies. In particular, controversy has focused on the question of whether distinct processes are responsible for canalization, the buffering against environmental or genetic variation, and for developmental stability, the buffering against random variation intrinsic in developmental processes. Here, we address this question for the size and shape of *Drosophila melanogaster* wings in an experimental design with extensively replicated and fully controlled genotypes. The amounts of variation among individuals and of fluctuating asymmetry differ markedly among genotypes, demonstrating a clear genetic basis for size and shape variability. For wing shape, there is a high correlation between the amounts of variation among individuals and fluctuating asymmetry, which indicates a correspondence between the two types of buffering. Likewise, the multivariate patterns of shape variation among individuals and of fluctuating asymmetry show a close association. For wing size, however, the amounts of individual variation and fluctuating asymmetry are not correlated. There was a significant link between the amounts of variation between wing size and shape, more so for fluctuating asymmetry than for variation among individuals. Overall, these experiments indicate a considerable degree of shared control of individual variation and fluctuating asymmetry, although it appears to differ between traits.

## Introduction

Developmental buffering is an important factor in evolutionary processes, because it can maintain adaptive phenotypic traits in the presence of genetic and environmental variation and it can conceal genetic variation from selection [1]–[3]. The processes responsible for developmental buffering are little known and have been debated extensively [4]–[6]. Possible mechanisms include molecular chaperone proteins such as Hsp90 [7] and the architecture of genetic regulatory networks responsible for gene expression [8], [9]. A particular focus in this debate is the question of how canalization, the buffering against genetic and environmental variation, is related to developmental stability, the buffering against random variation arising in developmental processes [4]. It has been contentious whether these are independent processes [4], [10] or whether they are manifestations of the same biological process [6]. Theoretical studies typically favor the latter point of view because both types of buffering emerge as results of developmental models [2], [11], and some authors treat the two concepts as synonymous [12]. Nevertheless, the relation of canalization and developmental stability is primarily an empirical question, and therefore needs to be addressed by studies of real organisms.

Empirical studies have tackled the question of whether canalization and developmental stability are distinct processes by comparing variation among individuals and the left-right asymmetries within individuals. Two main approaches have been used, which focus either on the amounts of variation or on covariance structures of multivariate features such as shape. Some studies have indicated that the amounts of individual variation and fluctuating asymmetry (FA) are correlated among genotypes [13], whereas others found no such association [14] and some studies reported differences according to traits [15]. Likewise, the studies comparing the multivariate patterns of shape variation have produced a range of results from strong congruence [16]–[18] to more or less complete independence [10], [19], [20], whereas other studies produced intermediate or mixed results [21], [22]. Many of these studies used population samples without controlling for genetic variation and with little replication, if any, and therefore these results should be interpreted with some caution.

This study used both these approaches simultaneously in the context of an experimental design with complete control of genetic variation, replicated for 115 distinct genotypes from the Exelixis deficiency stocks of *Drosophila melanogaster* [23]. Each of these strains carries a different deficiency on an otherwise isogenic background and therefore can be considered as a distinct and fully controlled genotype. We used the methods of geometric morphometrics [24]–[26] to quantify variation of size and shape in the wings of the flies. This design provided a large sample size for comparisons among genotypes and a high degree of replication for within-genotype analyses. The study yielded clear evidence for a common basis for developmental stability and canalization.

## Results

### Measurement Precision

**Figure 1:**
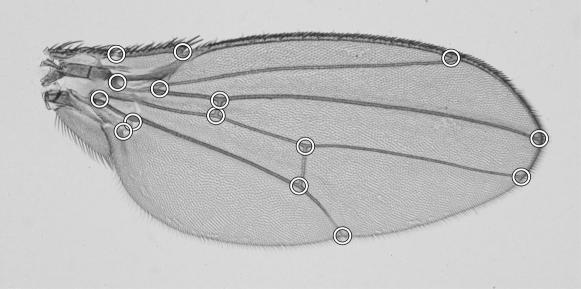
The set of 15 landmarks used in this study.

We digitized 15 landmarks on the left and right wings of each fly (Fig. 1). To estimate the amount of measurement error for shape, we carried out Procrustes ANOVA [16] for a subsample of 72 flies for which two images of each wing were taken and each image was digitized twice. The mean squares for FA and individual variation exceeded the error components by more than 41-fold (Table 1), indicating that measurement error was negligible relative to the biological shape variation. Likewise, measurement error for centroid size was negligible (not shown).

**Table 1:**
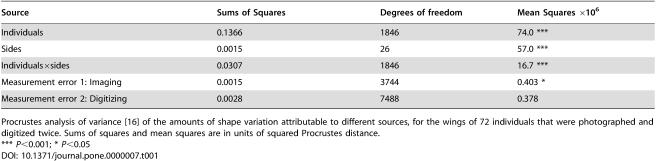
Analysis of measurement error

Source	Sums of Squares	Degrees of freedom	Mean Squares ×10^6^
Individuals	0.1366	1846	74.0 ***
Sides	0.0015	26	57.0 ***
Individuals×sides	0.0307	1846	16.7 ***
Measurement error 1: Imaging	0.0015	3744	0.403 *
Measurement error 2: Digitizing	0.0028	7488	0.378

Procrustes analysis of variance [16] of the amounts of shape variation attributable to different sources, for the wings of 72 individuals that were photographed and digitized twice. Sums of squares and mean squares are in units of squared Procrustes distance.

*** *P*<0.001; * *P*<0.05

### Amounts of Variation and Asymmetry

We used two different methods to quantify the amounts of shape variation among individuals and FA [27]. The first method uses Procrustes distance to quantify the absolute amount of shape variation and treats all aspects of shape variation equally, regardless of their degree of variability in the sample [27]. The second method is based on Mahalanobis distance and measures the amount of variation relative to the variability in the data set; features of shape that are relatively invariant are more heavily weighted, so that this measure can be interpreted as a measure of the degree to which shapes or shape asymmetries are unusual [27]. These two measures of shape variability were highly correlated with each other, both for individual variation (*r* = 0.81, *P*<0.0001) and for FA (*r* = 0.84, *P*<0.0001). Although the two measures are computed from different aspects of variation, they both convey similar information in the context of this study and therefore can be interpreted as nearly equivalent measures of shape variation.

The amounts of variation differed markedly among the different genotypes, although there was also a consistent effect of the vials in which the flies had been reared. For centroid size, the ANOVAs indicated that the variation among genotypes exceeded the variation among vials both for variation among individuals (*F*_114, 259_ = 2.08, *P*<0.0001) and for FA (*F*_114, 259_ = 2.26, *P*<0.0001). Similarly, the ANOVAs for both measures of shape variation indicated significant effects of the genotypes on variation among individuals (Procrustes distance: *F*_114, 259_ = 2.21, *P*<0.0001; Mahalanobis distance: *F*_114, 259_ = 2.63, *P*<0.0001) and on FA (Procrustes distance: *F*_114, 259_ = 2.34, *P*<0.0001; Mahalanobis distance: *F*_114, 259_ = 3.29, *P*<0.0001). These results show that the chromosomal deficiencies have clear effects on the amounts of variation among individuals and on FA, which in turn indicates a genetic basis for the amounts of variation.

### Relationship Between Variation Among Individuals and Fluctuating Asymmetry

**Figure 2:**
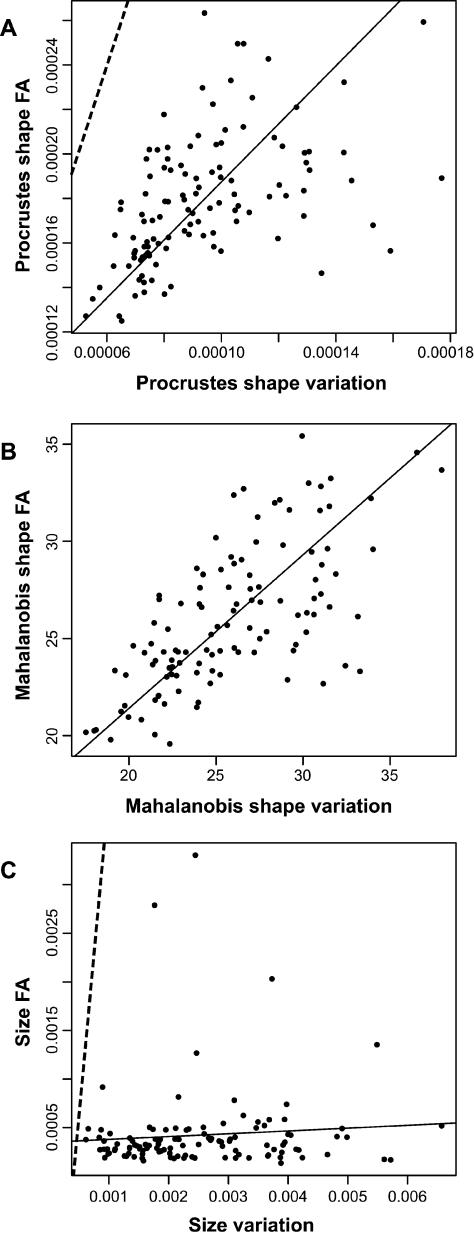
Relationships between individual variation and FA for shape and size. (A) Shape variation and FA quantified by Procrustes distance. (B) Shape variation and FA quantified using Mahalanobis distance. (C) Variation and FA of centroid size. The solid lines are major axis regression lines, and the dashed lines in (A) and (C) are the theoretical limits for the situation when left and right sides are independent (FA variance is 4 times the variance among individuals; see text for details).

For both measures of shape variability, the amounts of shape variation among individuals and of shape FA were significantly correlated across genotypes (Fig. 2A, B). In the analysis using Procrustes distance, the correlation was 0.49 (*P*<0.0001), and in the analysis using Mahalanobis distance, it was 0.67 (*P*<0.0001). Overall, there is a clear trend for genotypes with greater amounts of individual variation to have greater amounts of shape FA as well.

In contrast, the correlation between individual variation and FA of centroid size was low and not statistically significant (*r* = 0.074, *P* = 0.22; Fig. 2C). Unlike shape, therefore, there appears to be no connection between FA and individual variation of centroid size.

### Associations of Size and Shape Variation

**Figure 3:**
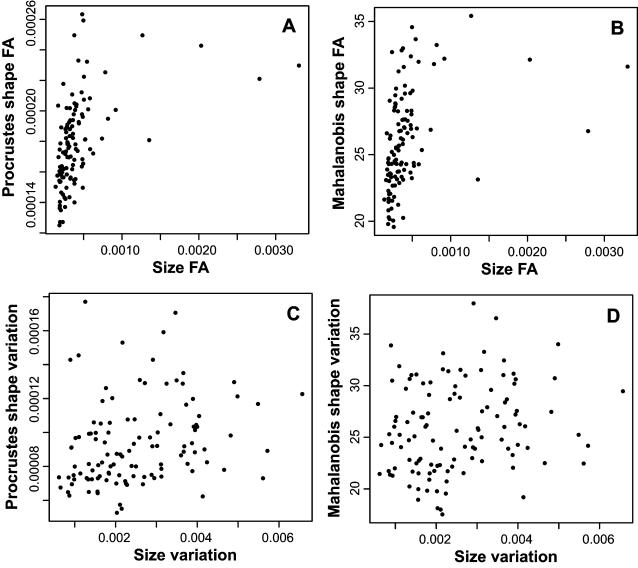
Relationships between size and shape for FA (A, B) and for individual variation (C, D).

The correlations between the amounts of FA of shape and of centroid size were 0.46 and 0.36 for the shape measures using Procrustes and Mahalanobis distances, respectively (Fig. 3A, B; both *P*<0.0001 in permutation tests). Accordingly, genotypes that are more asymmetric for size also tend to be more asymmetric for shape. The correlations between amounts of individual variation of size and shape were 0.25 (*P* = 0.0059) and 0.19 (*P* = 0.022) for the measures using Procrustes and Mahalanobis distances, respectively (Fig. 3C, D). The association between the amounts of size and shape variability therefore appeared to be present at both levels of variation, but was stronger for FA than for individual variation.

To assess the possibility that this association was caused by a direct developmental link between size and shape, we tested for allometry within genotypes by multivariate regression of shape on centroid size [28]–[30]. There was significant allometry among individuals in more than half of the genotypes (*P*<0.05 for 68 of the 115 genotypes after sequential Bonferroni adjustment) and size accounted for an average of 8.18% of shape variation among individuals. The asymmetry of size accounted for an average of 4.61% of the asymmetry of shape (*P*<0.05 for 14 genotypes after sequential Bonferroni adjustment). Accordingly, size accounts for only relatively minor proportions of shape variation and asymmetry under the conditions of our experiment. It therefore appears that the correlation between size and shape in the amounts asymmetry and individual variation is not simply the result from a direct allometric link between size and shape, but is based at least to a considerable part on linkages in the processes that produce or buffer against the variation.

### Patterns of Shape Variation

**Figure 4:**
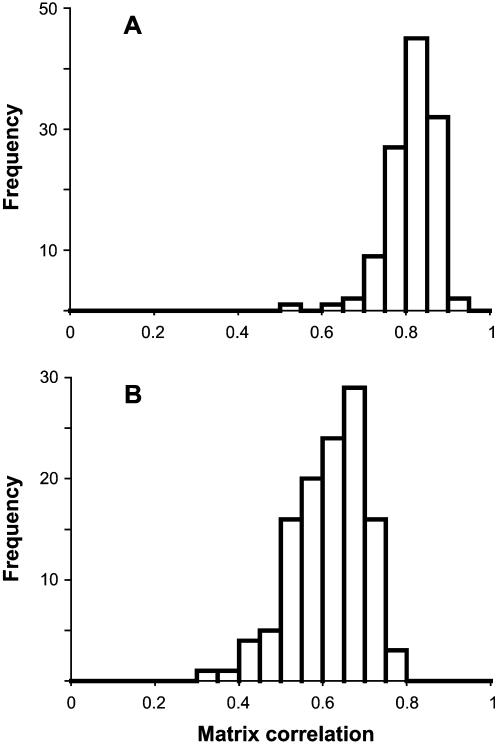
Matrix correlations between the covariance matrices for individual variation and FA. (A) Matrix correlations including the diagonal blocks (variances and covariances for *x* and *y* coordinates of each landmark). (B) Matrix correlations for covariance matrices without the diagonal blocks (only covariances among landmarks).

To examine whether among-individual variation and FA primarily concern the same or different features of shape, we quantified the degree of congruence between the respective patterns of covariation in landmark shifts. For this purpose, we computed matrix correlations between the respective covariance matrices for those 95 genotypes for which there were at least 50 specimens. Matrix correlations were computed both with the diagonal blocks included and excluded to examine whether the total patterns of landmark variation differ from the patterns of covariation among different landmarks [16]. The patterns of shape variation for FA and individual variation consistently showed a clear correspondence in all these genotypes. The matrix correlations ranged from 0.54 to 0.91 for the whole covariance matrices and from 0.31 to 0.79 if the diagonal blocks were omitted (Fig. 4). The difference in matrix correlations for the two methods of computation suggests that a component of the correspondence between individual variation and FA originated from the amounts of variation of individual landmarks. Nevertheless, because most matrix correlations were still fairly high even when the diagonal blocks of the covariance matrices were omitted, there appears to be a clear and consistent congruence between patterns of shape variation of individual variation and FA. The permutation tests indicated that all the matrix correlations were statistically significant (all *P* ≤ 0.0001).

## Discussion

This study shows a significant genetic effect on the amounts of individual variation and FA as well as a clear association in both the amounts and patterns of variation between individual variation and FA for wing shape. This is consistent with the idea of a common genetic and developmental basis for buffering of wing patterning processes against variation from different sources. The correlations between size and shape in the amounts of FA and individual variation provide further evidence in favor of a common basis for buffering. In contrast, the lack of association between the amounts of individual variation and FA for centroid size indicates that these relationships depend on the specific traits under study and the processes involved in their development. Here we discuss these findings and their implications for interpreting the mixed results of published empirical studies on canalization and developmental stability.

### Amounts of Variation and Asymmetry

Our data indicate a clear association of the amounts of individual variation and FA of wing shape across genotypes (Fig. 2A, B). This finding matches the results of an earlier study of sternopleural bristle counts in *Drosophila melanogaster* [13]. Moreover, experiments using overexpression of several genes in different regions of the *Drosophila* wing found an association between the severity of effects and levels of FA [31]. The result is also broadly consistent with a range of studies in other organisms that have found associations between individual variation and FA in comparisons across measurements [15], [20], [32], [33]. There are other studies, however, that did not find such a relationship [14] or where the results varied from trait to trait [15], [34]. Finally, comparisons among successive developmental stages in prenatal mice also revealed similar trends of FA and variation among individuals [35]. Whereas a relationship between the amounts of individual variation and FA seems to hold across genotypes and traits, there does not appear to be such an association between different stress regimes [15], [36].

In stark contrast to the shape data, the association between individual variation and FA did not hold in the analysis for centroid size (Fig. 2C). This different behavior, in the same experiment, suggests that different processes influence the amounts of variation of size and shape. It is conceivable that size and shape variation are subject to different sources of external variation. In particular, it is plausible that size variation is more sensitive to small variations in the availability and uptake of resources. The resulting differences in the acquired nutrients among individual larvae are likely to affect both sides jointly and therefore increase individual variation but not FA. Because the direct developmental links between size and shape are weak for most genotypes, size and shape can respond to such external factors differentially.

The discrepancy between these findings for size and shape highlights a methodological problem inherent in studies of developmental buffering: how can the effects of buffering be distinguished from differences in the initial input of developmental variation? Buffering is only observable if there is variation, and the resulting phenotypic variation is the joint expression of both the input of variation and the buffering of that variation by the developmental system. The original amount of variation, however, which is the input for the buffering processes in the developmental system, is unknown. The input of variation and buffering are therefore almost inextricably linked and cannot be separated without specifically designed experiments. Here we used samples of flies with controlled genotypes, so that genetic variation within samples can be ruled out. However, non-genetic effects cannot be controlled in this manner. The theoretical limit is a situation in which the environment is held constant so that the conditions under which the wings of two different flies develop are no more different than the conditions encountered by the two wings of the same fly. In this case, the left and right wings of individual flies would not be correlated, and the variance for individuals, var(0.5(right+left)), would be one-quarter of the variance for asymmetry, var(right−left). For shape, individual variation (quantified using Procrustes distance) exceeded this theoretical limit consistently, but only by relatively small amounts (Fig. 2A, dashed line). This suggests that the patterning processes determining shape are affected by micro-environmental heterogeneity only to a moderate degree. For centroid size, however, the among-individual variance far exceeds the theoretical minimum and, in all but a few genotypes, is much greater than FA (Fig. 2C), indicating that such environmental heterogeneity is a major factor for size variation. Accordingly, the lack of correlation among strains in the amounts of individual variation and FA for centroid size cannot be attributed unambiguously to an inherent difference between canalization and developmental stability, but must be due at least in part to a difference in the processes that generate developmental variation.

The correlation between the amounts of FA for centroid size and shape across the 115 genotypes exceeds the within-sample correlations of size and shape asymmetry for all but a few samples. Therefore, the direct developmental association of size and shape is not sufficient to account for the agreement of amounts of FA of size and shape. This is further evidence for a common genetic control of developmental variation of size and shape, although the data do not permit one to distinguish whether this control affects the origin of developmental noise or the developmental stability buffering against it. The association across many deficiency genotypes affecting different genomic regions may also be taken as evidence that a range of different genes contribute to the control of developmental stability, rather than just a few specialized genes [7], [37], [38], and is in agreement with theoretical arguments [2], [11] as well as other empirical evidence [39].

### Patterns of Shape Variation

We not only compared the amounts of variation, but also the patterns of landmark shifts associated with individual variation and FA. There is a close and consistent correspondence between the patterns of individual variation and FA. A similar correspondence of patterns of shape integration for individual variation and FA has been found previously in the wings of *Drosophila melanogaster* [17], interspecific hybrids of two *Drosophila* species [40], tsetse flies [16], and bumble bees [41]. In contrast, two studies in *Drosophila subobscura* found considerable differences [22], [42]. Just as for insect wings, a range of different results was also found for mammals. A good correspondence between individual variation and FA was reported for the mandibles of shrews [18] and mice [43], [44], whereas studies in the skulls of mice found no correspondence whatsoever [10] or only a weak but statistically significant association [45]. Small but significant matrix correlations were also found in a study of macaque skulls [20]. Finally, a study of the pharyngeal jaws of cichlid fish produced no significant matrix correlation, but there is the possibility that phenotypic plasticity contributed to this discrepancy [19]. Overall, there is no clear pattern discernible in these results, neither for the distribution across taxa nor for the organ systems that were studied. This lack of a consistent pattern has contributed to the contentious debate on the nature of canalization and developmental stability [4].

Because each of our samples was genetically uniform, we can rule out a contribution from allelic differences to the variation among individuals, which would produce effects that depend on the genetic composition of the sample and usually would differ from the within-individual effects. Imagine a population in which one locus with two alleles affects shape, so that the allelic differences will cause variation along a single line (with additive effect only) or in a plane (with additive and dominance effect). Unless the non-genetic components of variation also happen to be concentrated in the direction of this particular line or plane, the two components of variation will therefore be different. Even when more complex genetic models are used, the covariance structure among individuals will depend on the particular mix of genotypes, and may not reflect the inherent patterns of canalization. This reasoning can explain the closer resemblance of the patterns of FA to those of environmental rather than of genetic variation that has been found in empirical studies that specifically examined this effect [3], [22], [46]. Likewise, phenotypic plasticity in response to environmental differences, such as different trophic morphs [19], may introduce heterogeneity of covariance patterns that are unrelated to other patterns of variation. Because most studies were based on experimental designs that do not distinguish genetic and environmental components of variation, is not clear to which extent this reasoning also can explain the heterogeneous results in other comparisons of patterns of individual variation and FA.

Overall, the results of this study clearly indicate that both the amounts and the patterns of individual variation and FA of shape are associated consistently across a broad spectrum of distinct genotypes. This suggests that canalization and developmental stability for wing shape share a common basis [2], [6]. That these relationships emerged consistently across a large sample of different genotypes agrees with the view that buffering and its genetic control may be an intrinsic property of developmental systems [9], [11]. The difference between the results for shape and size, however, underscores that developmental buffering is specific to the traits and the processes involved in their development [31]. Developmental stability and canalization therefore need to be considered in the specific context of the traits under study.

## Materials and Methods

### Flies and Measurements

The flies used here were offspring from crosses between the Exelixis deficiency stocks [23] and the strain with the common genetic background used for generating all the deficiency stocks (strain numbers and the statistics that form the basis of this study can be found in Table S1). Accordingly, the flies were isogenic, except for the small genomic regions of the deficiencies themselves, for which the flies are hemizygous, and the flanking sequences from the transposable element insertion used to produce the deficiencies [23]. The flies were reared in vials of cornmeal-melasses fly food at 25°C and killed one to two days after emergence. The wings were mounted on slides and digital images of the wings were taken with a Leica DFC320 camera attached to a Leica DM LB2 compound microscope.

A set of 15 landmarks was digitized on each image (Fig. 1). To assess the amount of error due to the imaging and digitizing steps, two different images of each wing were taken for a subsample of 72 flies and each of these images was digitized twice. The remaining analyses used samples from 115 lines, averaging 62 flies per strain (ranging from 40 to 102), for a total of 7123 flies (for variation among individuals) or 7046 flies (for FA). For each strain, flies from multiple vials were used, ranging from two to ten vials per strain, with an average of 19 flies per vial (varying with the sample size used and on the fecundity and viability of the flies).

### Statistical Analyses

The shape information was extracted from the landmark coordinates with a generalized least-squares Procrustes fit [25]. The measurement error components for shape were quantified with Procrustes ANOVA [16], [19] for the subsample of flies for which replicate images had been taken.

To quantify individual variation and FA of wing size, we used the within-sample variance of the centroid size [25] of the wings and the variance of the (right−left) difference of centroid size. This procedure corresponds to the two-factor ANOVA model customary in asymmetry studies [47] and automatically corrects for the presence of directional asymmetry.

We used two different methods to quantify variation, which are based on different measures of morphological distance: Procrustes distance and Mahalanobis distance [27]. Procrustes distance is a measure of absolute shape differences [25] and treats shape deviations from the sample mean equally, regardless of their direction. Procrustes variances were obtained by summing the squared deviations from the respective sample means and dividing by the appropriate degrees of freedom. Mahalanobis distance is a measure of distance relative to the variation in each direction of the multivariate space [48]. We used the pooled within-group covariance matrix (within genotypes and sexes) to compute the Mahalanobis distance of each observation from the mean shape of its group [27]. A measure of the amount of variation within samples was obtained by summing up the squares of the Mahalanobis distances and dividing by the respective degrees of freedom.

To test whether the amounts of variation differed among genotypes, we used an extension of Levene's test [49], that is, an ANOVA of the individual deviations from the respective group averages. To take into account the effect of the environment in which the flies were reared, we used a nested ANOVA design with vials nested within genotypes. The tests used the vial effect as the error term, and a significant result therefore indicates that the differences among genotypes exceed the environmental variation among vials. The extension for the shape data was based on the fact that the Procrustes and Mahalanobis distances of individual observations from the sample mean shapes are measures of deviation that are similar to the absolute value of the deviation from the mean of scalar variables. Accordingly, the test used the same nested ANOVA of these Procrustes or Mahalanobis distances, with the genotypes as the grouping criterion and vials as the error term.

To examine the correspondence between the amounts of individual variation and FA, we computed the variances based on the two distance measures for data sets with either the mean shapes of both wings or the signed (right−left) differences of wing shape. Product-moment correlations were then computed across genotypes. The statistical significance of correlations was assessed with permutation tests [50] with 10,000 random permutations per test.

Allometry within genotypes was tested by multivariate regression of shape on centroid size [28], [29]. This was done for both the variation among individuals (means of both sides for size and shape) as well as for FA (signed asymmetry values of size and shape). Percentages of shape variation for which size accounted were computed from the Procrustes variances of the shapes predicted by the regression and the total Procrustes variance for the respective analysis (asymmetry or means of both sides). The statistical significance of the regressions was established with permutation tests with 10,000 random permutations per analysis.

For the strains for which at least 50 specimens were available, we also compared the patterns of shape variation between individual variation and FA [16]. Matrix correlations between the covariance matrices for the means of both wings of each individual and for the signed (right−left) differences were computed and tested with a matrix permutation test using 10,000 random permutations of landmarks in one of the matrices (*x* and *y* coordinates of each landmark were kept together) [16]. Matrix correlations were computed both with and without the diagonal blocks of the covariance matrices (variances of landmark coordinates and covariances between *x* and *y* coordinates of each landmark) [16].

## Supporting Information

Table S1 Strains used in this study and various sample statistics.
(0.36 MB DOC)
