# Supplementary material for: A Single Basis for Developmental Buffering of Drosophila Wing Shape
Source: PLoS One. 2006 Dec 20;1(1):e7. doi: 10.1371/journal.pone.0000007 (PMC1762351; doi:10.1371/journal.pone.0000007)
Supplement: Table S1 — Strains used in this study and various sample statistics. (0.36 MB DOC) [file pone.0000007.s001.doc]

1. Exelixis strain number

2. Shape variance (based on Procrustes distances)

3. Shape variance (based on Mahalanobis distances)

4. Centroid size variance

5. Shape FA (based on Procrustes distances)

6. Shape FA (based on Mahalanobis distances)

7. Size FA

8. Percentage of shape variance (Procrustes metric) accounted for by allometry

9. P-value of the permutation test for allometry

10. Percentage of shape asymmetry (Procrustes metric) accounted for by asymmetry of centroid size

11. P-value for permutation test for association between asymmetries of shape and of centroid size

12. Matrix correlation (diagonal blocks included) between shape variation among individuals and asymmetry

13. P-value for matrix permutation test (diagonal blocks included) between shape variation among individuals and asymmetry

14. Matrix correlation (diagonal blocks excluded) between shape variation among individuals and asymmetry

15 P-value for matrix permutation test (diagonal blocks included) between shape variation among individuals and asymmetry

| 1 | 2 | 3 | 4 | 5 | 6 | 7 | 8 | 9 | 10 | 11 | 12 | 13 | 14 | 15 |
| --- | --- | --- | --- | --- | --- | --- | --- | --- | --- | --- | --- | --- | --- | --- |
| 6051 | 8.59E-05 | 31.73968 | 0.002162 | 0.000195 | 33.3856 | 0.000817 | 8.133622 | 0 | 4.365301 | 0.0157 | 0.827992 | 0 | 0.650664 | 0 |
| 6052 | 9.19E-05 | 28.55943 | 0.003684 | 0.000208 | 32.12979 | 0.000583 | 9.270871 | 0.0015 | 7.718251 | 0.0007 | 0.81699 | 0 | 0.638624 | 0 |
| 6053 | 0.000143 | 34.05532 | 0.000893 | 0.000201 | 32.39357 | 0.000917 | 6.490004 | 0.021 | 7.307081 | 0.0008 | 0.663175 | 0 | 0.415936 | 0 |
| 6054 | 0.000146 | 32.11524 | 0.001095 | 0.000188 | 28.45567 | 0.000266 | 4.037889 | 0.0696 | 3.0273 | 0.1641 | 0.856746 | 0 | 0.688804 | 0 |
| 6055 | 8.92E-05 | 28.85917 | 0.002334 | 0.000168 | 27.10167 | 0.000485 | 6.206562 | 0.004 | 2.796809 | 0.2254 | 0.833305 | 0 | 0.604129 | 0 |
| 6056 | 0.000106 | 30.59335 | 0.001857 | 0.00025 | 33.34639 | 0.00038 | 4.534711 | 0.0553 | 2.627026 | 0.3118 | 0.889906 | 0 | 0.772788 | 0 |
| 6057 | 7.77E-05 | 27.67799 | 0.001437 | 0.000202 | 31.43351 | 0.000322 | 2.518822 | 0.3863 | 4.70109 | 0.0451 | 0.812428 | 0 | 0.62236 | 0 |
| 6058 | 0.000097 | 29.09551 | 0.002579 | 0.000222 | 29.95213 | 0.000504 | 11.91751 | 0 | 2.999788 | 0.1714 | 0.801311 | 0 | 0.576494 | 0 |
| 6059 | 7.48E-05 | 26.20785 | 0.000973 | 0.000202 | 29.08241 | 0.000281 | 11.57676 | 0 | 3.667825 | 0.1003 | 0.760287 | 0 | 0.497309 | 0 |
| 6060 | 9.34E-05 | 29.49408 | 0.002445 | 0.00023 | 31.81856 | 0.003303 | 17.16087 | 0 | 9.989495 | 0.0007 | 0.813312 | 0 | 0.677452 | 0 |
| 6061 | 9.12E-05 | 30.74909 | 0.000919 | 0.000189 | 29.77007 | 0.000279 | 4.909254 | 0.0448 | 7.042029 | 0.0029 | 0.734244 | 0 | 0.445642 | 0 |
| 6062 | 0.00008 | 25.87482 | 0.002497 | 0.000194 | 25.8531 | 0.000326 | 12.08954 | 0 | 3.018337 | 0.2115 | 0.789147 | 0 | 0.600283 | 0 |
| 6063 | 7.35E-05 | 25.50054 | 0.000848 | 0.000182 | 28.78742 | 0.000272 | 1.866031 | 0.4732 | 8.015938 | 0.0001 | 0.832468 | 0 | 0.610257 | 0 |
| 6064 | 6.28E-05 | 21.61677 | 0.000867 | 0.000164 | 23.87011 | 0.000477 | 5.10714 | 0.0305 | 3.413672 | 0.1437 | 0.791662 | 0 | 0.526046 | 0 |
| 6066 | 9.73E-05 | 25.52644 | 0.001107 | 0.000158 | 24.53268 | 0.000281 | 2.203896 | 0.4853 | 5.379525 | 0.0201 | 0.747654 | 0 | 0.467772 | 0 |
| 6067 | 0.000143 | 38.30116 | 0.002913 | 0.000232 | 33.93053 | 0.000545 | 6.771333 | 0.0002 | 6.836727 | 0 | 0.790684 | 0 | 0.493332 | 0 |
| 6069 | 7.38E-05 | 24.26418 | 0.002702 | 0.000198 | 27.76787 | 0.000372 | 7.707774 | 0.0024 | 7.620523 | 0.003 | 0.816308 | 0 | 0.588994 | 0 |
| 6070 | 8.23E-05 | 27.26537 | 0.001671 | 0.000193 | 28.51671 | 0.000316 | 10.12458 | 0 | 1.814638 | 0.6967 | 0.781283 | 0 | 0.54743 | 0 |
| 6071 | 9.29E-05 | 27.64363 | 0.00104 | 0.000181 | 26.31977 | 0.000269 | 3.052337 | 0.2821 | 1.214931 | 0.9422 | 0.766053 | 0 | 0.591654 | 0 |
| 6073 | 0.000119 | 30.53757 | 0.001679 | 0.000207 | 26.52126 | 0.000504 | 9.415017 | 0.0047 | 3.338362 | 0.2237 | 0.751423 | 0 | 0.532586 | 0 |
| 6076 | 0.000112 | 31.82221 | 0.001417 | 0.000217 | 29.90776 | 0.000372 | 4.506384 | 0.0488 | 6.291425 | 0.0509 | 0.84067 | 0 | 0.583417 | 0 |
| 6079 | 8.12E-05 | 22.03124 | 0.001953 | 0.000198 | 27.51477 | 0.000479 | 11.68672 | 0.0013 | 10.35845 | 0 | 0.760985 | 0 | 0.545415 | 0 |
| 6083 | 7.99E-05 | 26.80414 | 0.001003 | 0.000218 | 33.00578 | 0.000237 | 4.272468 | 0.0077 | 4.013715 | 0.0132 | 0.876372 | 0 | 0.67838 | 0 |
| 6084 | 9.98E-05 | 31.27055 | 0.001513 | 0.00019 | 28.97595 | 0.000285 | 8.05422 | 0.0002 | 5.541935 | 0.0031 | 0.800834 | 0 | 0.556795 | 0 |
| 6085 | 0.000087 | 27.56159 | 0.001803 | 0.00018 | 30.30616 | 0.000289 | 2.168474 | 0.2186 | 4.676866 | 0.0022 | 0.809479 | 0 | 0.510902 | 0 |
| 6086 | 8.15E-05 | 24.12675 | 0.00376 | 0.000163 | 28.97355 | 0.000276 | 9.053891 | 0.0001 | 2.177758 | 0.2211 | 0.760808 | 0 | 0.5577 | 0 |
| 6087 | 7.31E-05 | 26.79918 | 0.001295 | 0.000158 | 29.27872 | 0.000207 | 7.155132 | 0.0001 | 2.910479 | 0.0512 | 0.854238 | 0 | 0.632331 | 0 |
| 6088 | 0.000129 | 31.19219 | 0.002717 | 0.000201 | 31.92358 | 0.000389 | 4.468806 | 0.0191 | 3.343329 | 0.0463 | 0.745113 | 0 | 0.516862 | 0 |
| 6089 | 9.61E-05 | 22.64163 | 0.001514 | 0.000176 | 23.3179 | 0.000302 | 7.163939 | 0.0004 | 3.72345 | 0.0247 | 0.876063 | 0 | 0.73747 | 0 |
| 6090 | 5.27E-05 | 18.23661 | 0.002035 | 0.000127 | 20.46987 | 0.000194 | 11.4287 | 0 | 2.262743 | 0.0405 | 0.875326 | 0 | 0.698686 | 0 |
| 6091 | 9.94E-05 | 31.25621 | 0.001393 | 0.000194 | 33.1617 | 0.000362 | 2.920973 | 0.1059 | 5.275168 | 0.0014 | 0.739671 | 0 | 0.39549 | 0 |
| 6092 | 6.76E-05 | 24.54229 | 0.000652 | 0.00015 | 28.61958 | 0.000489 | 4.222089 | 0.0108 | 7.55438 | 0 | 0.846553 | 0 | 0.646418 | 0 |
| 6093 | 8.87E-05 | 26.23899 | 0.003666 | 0.000164 | 26.56262 | 0.000218 | 5.121694 | 0.0046 | 4.826688 | 0.0019 | 0.76781 | 0 | 0.514843 | 0 |
| 6094 | 7.51E-05 | 23.23265 | 0.00288 | 0.00019 | 27.00078 | 0.000352 | 8.45638 | 0.0001 | 5.384842 | 0.0028 | 0.887485 | 0 | 0.725116 | 0 |
| 6095 | 0.000105 | 27.72257 | 0.003974 | 0.000182 | 27.04443 | 0.000742 | 15.2507 | 0 | 4.134015 | 0.0265 | 0.713665 | 0 | 0.512225 | 0 |
| 6096 | 6.95E-05 | 21.49017 | 0.000932 | 0.000153 | 24.95382 | 0.000192 | 2.599595 | 0.162 | 4.521704 | 0.0054 | 0.866923 | 0 | 0.6899 | 0 |
| 6097 | 7.36E-05 | 21.63498 | 0.000615 | 0.000155 | 26.05024 | 0.000378 | 2.617279 | 0.1431 | 3.142491 | 0.0357 | 0.764862 | 0 | 0.485948 | 0 |
| 6098 | 6.48E-05 | 21.90773 | 0.000837 | 0.000175 | 27.24854 | 0.0004 | 3.136536 | 0.0519 | 3.951791 | 0.007 | 0.877471 | 0 | 0.717066 | 0 |
| 6099 | 9.21E-05 | 26.04475 | 0.00302 | 0.000185 | 29.42362 | 0.000363 | 9.827668 | 0 | 3.184535 | 0.0326 | 0.834412 | 0 | 0.643876 | 0 |
| 6100 | 0.000153 | 33.40122 | 0.002172 | 0.000168 | 26.34753 | 0.00038 | 2.058752 | 0.2691 | 3.551821 | 0.0233 | 0.632843 | 0 | 0.438501 | 0 |
| 6101 | 7.19E-05 | 20.05791 | 0.00178 | 0.000152 | 23.35888 | 0.000383 | 11.27336 | 0 | 3.426463 | 0.0309 | 0.782856 | 0 | 0.593585 | 0 |
| 6102 | 7.23E-05 | 22.12968 | 0.000989 | 0.000145 | 24.54209 | 0.000232 | 5.893799 | 0.0032 | 4.06145 | 0.0097 | 0.80835 | 0 | 0.528787 | 0 |
| 6103 | 8.12E-05 | 25.16922 | 0.003112 | 0.000203 | 30.36088 | 0.000404 | 10.9693 | 0 | 10.57178 | 0.0005 | 0.823511 | 0 | 0.683379 | 0 |
| 6104 | 8.07E-05 | 22.91085 | 0.001577 | 0.000158 | 23.22199 | 0.00016 | 8.998142 | 0.0002 | 7.593118 | 0.0005 | 0.759861 | 0 | 0.573443 | 0 |
| 6105 | 0.000074 | 22.88193 | 0.003122 | 0.000161 | 24.56593 | 0.000188 | 7.631618 | 0.003 | 2.254948 | 0.0464 | 0.883627 | 0 | 0.727612 | 0 |
| 6106 | 7.31E-05 | 24.14906 | 0.002947 | 0.000138 | 21.61953 | 0.000191 | 2.968201 | 0.0035 | 2.440014 | 0.0096 | 0.854607 | 0 | 0.697839 | 0 |
| 6107 | 0.000177 | 29.87194 | 0.001258 | 0.000189 | 26.38064 | 0.000208 | 7.898892 | 0.0034 | 5.067596 | 0.0001 | 0.709435 | 0 | 0.542912 | 0 |
| 6108 | 7.63E-05 | 21.04706 | 0.001829 | 0.000162 | 24.42161 | 0.000353 | 6.330681 | 0.0001 | 2.158215 | 0.1117 | 0.890465 | 0 | 0.739369 | 0 |
| 6109 | 7.59E-05 | 24.19791 | 0.001136 | 0.00017 | 26.98329 | 0.000332 | 5.96523 | 0.0011 | 6.063553 | 0.0007 | 0.890378 | 0 | 0.693501 | 0 |
| 6110 | 0.000111 | 31.78172 | 0.003096 | 0.000225 | 32.03535 | 0.000783 | 5.663968 | 0.0001 | 2.618467 | 0.0424 | 0.842058 | 0 | 0.699206 | 0 |
| 6111 | 9.41E-05 | 26.22069 | 0.001736 | 0.000263 | 32.54753 | 0.000483 | 6.192954 | 0 | 5.032468 | 0.0009 | 0.914235 | 0 | 0.785287 | 0 |
| 6112 | 9.92E-05 | 30.85747 | 0.001349 | 0.000178 | 27.23829 | 0.000394 | 1.097067 | 0.5135 | 3.588606 | 0.0021 | 0.89296 | 0 | 0.720197 | 0 |
| 6113 | 6.52E-05 | 19.09191 | 0.001558 | 0.000125 | 19.88914 | 0.000186 | 4.754232 | 0.0003 | 1.928464 | 0.0487 | 0.857216 | 0 | 0.683016 | 0 |
| 6114 | 0.000129 | 28.1047 | 0.003248 | 0.000172 | 25.55092 | 0.000626 | 3.514514 | 0.0623 | 5.813491 | 0.0001 | 0.728895 | 0 | 0.535519 | 0 |
| 6115 | 0.00013 | 30.9191 | 0.004904 | 0.000196 | 28.22678 | 0.000491 | 9.227065 | 0 | 2.996097 | 0.0323 | 0.766499 | 0 | 0.590514 | 0 |
| 6116 | 0.000101 | 30.89688 | 0.003945 | 0.000211 | 26.46613 | 0.000352 | 6.871983 | 0.0001 | 3.281749 | 0.0361 | 0.852195 | 0 | 0.664486 | 0 |
| 6117 | 7.72E-05 | 22.1883 | 0.003882 | 0.00015 | 21.78273 | 0.000137 | 8.892096 | 0 | 2.77736 | 0.0683 | 0.859748 | 0 | 0.694896 | 0 |
| 6118 | 0.00009 | 26.24618 | 0.004179 | 0.000173 | 24.70036 | 0.000271 | 8.672114 | 0 | 2.625935 | 0.0727 | 0.831794 | 0 | 0.55857 | 0 |
| 6119 | 9.82E-05 | 27.69738 | 0.004816 | 0.000204 | 27.85056 | 0.000408 | 7.105067 | 0.0011 | 5.566869 | 0.0001 | 0.816376 | 0 | 0.652013 | 0 |
| 6120 | 5.75E-05 | 18.12992 | 0.002109 | 0.00014 | 20.42575 | 0.000381 | 7.155103 | 0 | 2.780699 | 0.0229 | 0.835748 | 0 | 0.623062 | 0 |
| 6121 | 6.23E-05 | 19.33798 | 0.004136 | 0.00015 | 23.55741 | 0.000278 | 13.38578 | 0 | 4.840758 | 0.0011 | 0.825837 | 0 | 0.582339 | 0 |
| 6122 | 0.000073 | 22.63 | 0.005609 | 0.00017 | 24.04062 | 0.000175 | 12.98049 | 0 | 3.458518 | 0.0112 | 0.838169 | 0 | 0.65537 | 0 |
| 6123 | 0.000073 | 21.67139 | 0.001568 | 0.000142 | 21.93161 | 0.000264 | 9.797714 | 0.0006 | 3.336115 | 0.0646 | 0.790623 | 0 | 0.605926 | 0 |
| 6124 | 6.99E-05 | 20.89824 | 0.002255 | 0.000136 | 20.9636 | 0.000188 | 6.972372 | 0.0001 | 4.157305 | 0.0057 | 0.861028 | 0 | 0.692797 | 0 |
| 6125 | 8.01E-05 | 22.49283 | 0.001876 | 0.000137 | 19.70485 | 0.00026 | 12.77278 | 0 | 4.202617 | 0.005 | 0.82812 | 0 | 0.654854 | 0 |
| 6126 | 5.51E-05 | 17.68729 | 0.00215 | 0.000135 | 20.32607 | 0.000206 | 14.48761 | 0 | 2.670065 | 0.0766 | 0.875132 | 0 | 0.699128 | 0 |
| 6127 | 7.86E-05 | 27.80997 | 0.003121 | 0.000172 | 25.12071 | 0.000263 | 6.017429 | 0.0003 | 3.34358 | 0.0446 | 0.793466 | 0 | 0.56332 | 0 |
| 6128 | 6.95E-05 | 21.70747 | 0.002668 | 0.000155 | 24.01107 | 0.000394 | 10.23618 | 0 | 5.936084 | 0 | 0.865485 | 0 | 0.690387 | 0 |
| 6129 | 0.000131 | 27.36547 | 0.003494 | 0.000201 | 27.20549 | 0.00056 | 13.05147 | 0 | 9.80648 | 0 | 0.833536 | 0 | 0.731891 | 0 |
| 6130 | 9.99E-05 | 29.36803 | 0.001826 | 0.000156 | 23.06013 | 0.000331 | 11.85686 | 0 | 4.910321 | 0.015 | 0.777844 | 0 | 0.606269 | 0 |
| 6131 | 0.000108 | 30.19283 | 0.002467 | 0.00025 | 35.66543 | 0.001267 | 5.413029 | 0.0055 | 18.36331 | 0.0002 | 0.818074 | 0 | 0.744584 | 0 |
| 6132 | 6.43E-05 | 20.14302 | 0.00156 | 0.000127 | 21.12716 | 0.00023 | 10.73259 | 0 | 3.166481 | 0.0349 | 0.803615 | 0 | 0.565957 | 0 |
| 6133 | 0.000105 | 27.11795 | 0.001638 | 0.00017 | 25.75316 | 0.000291 | 5.473995 | 0.0059 | 2.415354 | 0.15 | 0.748205 | 0 | 0.523383 | 0 |
| 6134 | 0.000117 | 25.40412 | 0.005484 | 0.000181 | 23.2813 | 0.001354 | 20.37137 | 0 | 3.70279 | 0.0188 | 0.779655 | 0 | 0.627654 | 0 |
| 6135 | 8.24E-05 | 24.17919 | 0.004237 | 0.00014 | 21.8534 | 0.000192 | 10.62803 | 0 | 2.604847 | 0.0885 | 0.794308 | 0 | 0.601001 | 0 |
| 6136 | 8.03E-05 | 22.32227 | 0.00239 | 0.000179 | 23.18141 | 0.000199 | 5.410599 | 0.0007 | 1.891626 | 0.2137 | 0.82924 | 0 | 0.666796 | 0 |
| 6137 | 0.00012 | 31.44062 | 0.003894 | 0.000162 | 22.85079 | 0.000245 | 16.02685 | 0 | 3.986303 | 0.014 | 0.760044 | 0 | 0.567673 | 0 |
| 6138 | 9.71E-05 | 24.97816 | 0.002844 | 0.000165 | 23.45346 | 0.000363 | 13.27703 | 0 | 3.233849 | 0.016 | 0.78419 | 0 | 0.600114 | 0 |
| 6139 | 7.48E-05 | 22.48897 | 0.00209 | 0.000154 | 23.62324 | 0.000184 | 7.027133 | 0 | 4.080527 | 0.0011 | 0.909726 | 0 | 0.730532 | 0 |
| 6261 | 0.0001 | 25.97383 | 0.003033 | 0.000205 | 27.85801 | 0.000383 | 15.14096 | 0 | 3.665911 | 0.0075 | 0.853924 | 0 | 0.702148 | 0 |
| 6262 | 9.19E-05 | 24.23383 | 0.002901 | 0.00017 | 23.88778 | 0.000309 | 15.21974 | 0 | 4.475834 | 0.0026 | 0.737027 | 0 | 0.523231 | 0 |
| 6279 | 7.25E-05 | 21.86018 | 0.001422 | 0.000154 | 22.17474 | 0.000199 | 7.506465 | 0 | 2.66444 | 0.0553 | 0.867452 | 0 | 0.715189 | 0 |
| 6283 | 0.000065 | 20.48835 | 0.001343 | 0.000178 | 24.78788 | 0.000289 | 3.048483 | 0.2615 | 6.690346 | 0.0044 | 0.837422 | 0 | 0.554586 | 0 |
| 6285 | 0.000121 | 34.29179 | 0.004985 | 0.000204 | 29.79846 | 0.000404 | 19.9067 | 0 | 2.93581 | 0.0121 | 0.826286 | 0 | 0.650317 | 0 |
| 7208 | 0.000129 | 31.30142 | 0.003643 | 0.000184 | 27.53397 | 0.000319 | 12.10495 | 0 | 3.338139 | 0.0547 | 0.784007 | 0 | 0.614948 | 0 |
| 7210 | 8.65E-05 | 25.00871 | 0.0036 | 0.000181 | 24.32771 | 0.000522 | 9.458826 | 0 | 5.472783 | 0.0034 | 0.807487 | 0 | 0.501328 | 0 |
| 7253 | 0.000126 | 26.38562 | 0.001765 | 0.000221 | 27.02337 | 0.002788 | 10.15789 | 0.0002 | 33.06946 | 0 | 0.801205 | 0 | 0.596166 | 0 |
| 7315 | 0.000078 | 22.67968 | 0.004656 | 0.00016 | 23.6947 | 0.000222 | 10.29075 | 0 | 2.970557 | 0.0345 | 0.84743 | 0 | 0.6499 | 0 |
| 7317 | 0.00011 | 26.40304 | 0.004043 | 0.000174 | 24.51769 | 0.000422 | 7.654914 | 0.0004 | 4.326164 | 0.0132 | 0.771063 | 0 | 0.530199 | 0 |
| 7357 | 0.000159 | 33.42517 | 0.003171 | 0.000156 | 23.5147 | 0.000225 | 25.41155 | 0 | 1.158372 | 0.7674 | 0.673515 | 0 | 0.464921 | 0 |
| 8098 | 0.000131 | 31.76076 | 0.002592 | 0.000193 | 26.81498 | 0.000437 | 4.037478 | 0.032 | 2.432227 | 0.1516 | 0.803869 | 0 | 0.543369 | 0 |
| 8101 | 0.000135 | 32.62354 | 0.003658 | 0.000147 | 23.80494 | 0.000209 | 5.019905 | 0.0366 | 8.015615 | 0 | 0.537065 | 0 | 0.312795 | 0 |
| 8104 | 8.14E-05 | 23.00827 | 0.001344 | 0.000179 | 24.45658 | 0.000297 | 4.985689 | 0.0025 | 4.358537 | 0.0051 | 0.88092 | 0 | 0.733913 | 0 |
| 9000 | 0.000171 | 36.82786 | 0.003465 | 0.000259 | 34.80553 | 0.000497 | 12.33168 | 0 | 4.771662 | 0.0039 | 0.775685 | 0 | 0.586897 | 0 |
| 9001 | 6.92E-05 | 21.85072 | 0.00228 | 0.000162 | 22.21349 | 0.000215 | 3.805403 | 0.0088 | 4.913724 | 0.0005 | 0.890407 | 0 | 0.748253 | 0 |
| 9002 | 7.43E-05 | 23.0078 | 0.002437 | 0.000158 | 22.49653 | 0.00019 | 9.446724 | 0 | 2.588525 | 0.0597 | 0.842453 | 0 | 0.631092 | 0 |
| 9003 | 8.84E-05 | 27.52241 | 0.003942 | 0.000175 | 24.40566 | 0.000586 | 11.01042 | 0 | 3.28361 | 0.0797 | 0.838785 | 0 | 0.679915 | 0 |
| 9004 | 8.74E-05 | 22.37205 | 0.002028 | 0.000191 | 25.71251 | 0.00029 | 8.505954 | 0 | 2.168135 | 0.1886 | 0.821618 | 0 | 0.647456 | 0 |
| 9005 | 7.23E-05 | 24.90144 | 0.00133 | 0.000173 | 25.35607 | 0.000234 | 8.040295 | 0 | 3.686416 | 0.0306 | 0.841597 | 0 | 0.62782 | 0 |
| 9006 | 7.43E-05 | 23.0991 | 0.001615 | 0.000156 | 24.05258 | 0.000327 | 3.636055 | 0.0396 | 5.736296 | 0.0006 | 0.833897 | 0 | 0.587423 | 0 |
| 9007 | 9.11E-05 | 24.68783 | 0.000918 | 0.000182 | 24.61065 | 0.000362 | 3.910912 | 0.0359 | 3.684275 | 0.0411 | 0.859445 | 0 | 0.698303 | 0 |
| 9008 | 0.000105 | 29.79565 | 0.003342 | 0.000175 | 24.8655 | 0.00019 | 6.508252 | 0.0044 | 3.324945 | 0.031 | 0.822437 | 0 | 0.648311 | 0 |
| 9009 | 0.000108 | 31.68188 | 0.002296 | 0.000212 | 29.83743 | 0.000474 | 4.786963 | 0.0083 | 3.258583 | 0.0534 | 0.893659 | 0 | 0.77928 | 0 |
| 9011 | 8.71E-05 | 24.08769 | 0.002119 | 0.000165 | 23.40338 | 0.000495 | 6.396862 | 0 | 2.05101 | 0.0978 | 0.789077 | 0 | 0.550599 | 0 |
| 9017 | 6.99E-05 | 19.944 | 0.002019 | 0.000156 | 21.76221 | 0.000239 | 6.968368 | 0 | 5.639127 | 0.0009 | 0.878775 | 0 | 0.729441 | 0 |
| 9028 | 9.37E-05 | 23.43061 | 0.003869 | 0.000163 | 24.46443 | 0.000195 | 12.60502 | 0 | 2.191592 | 0.217 | 0.823519 | 0 | 0.619092 | 0 |
| 9034 | 8.92E-05 | 24.37477 | 0.005719 | 0.000204 | 26.75896 | 0.000168 | 14.12799 | 0 | 2.988813 | 0.0307 | 0.855523 | 0 | 0.658694 | 0 |
| 9045 | 0.000123 | 29.63391 | 0.006571 | 0.000181 | 24.50144 | 0.000519 | 19.99488 | 0 | 5.376129 | 0.0014 | 0.766428 | 0 | 0.595791 | 0 |
| 9046 | 0.000116 | 28.89398 | 0.00373 | 0.000243 | 32.29953 | 0.00203 | 13.34993 | 0 | 5.419895 | 0.0045 | 0.791313 | 0 | 0.620509 | 0 |
| 9048 | 0.000106 | 25.32347 | 0.001466 | 0.000177 | 25.82452 | 0.000314 | 9.284925 | 0 | 2.882852 | 0.0268 | 0.873638 | 0 | 0.70513 | 0 |
| 9057 | 0.000104 | 30.49385 | 0.003915 | 0.000188 | 25.48034 | 0.000322 | 5.095738 | 0.0006 | 2.592524 | 0.0396 | 0.8758 | 0 | 0.689433 | 0 |
| 9058 | 7.57E-05 | 21.61466 | 0.001838 | 0.000143 | 20.19692 | 0.000253 | 2.061372 | 0.1514 | 2.426078 | 0.0653 | 0.886359 | 0 | 0.66549 | 0 |
| 9061 | 0.00012 | 24.85639 | 0.00192 | 0.000186 | 22.88088 | 0.000274 | 7.203224 | 0.0016 | 8.773039 | 0 | 0.814997 | 0 | 0.647379 | 0 |
| 9065 | 9.67E-05 | 27.25814 | 0.001021 | 0.000192 | 27.72495 | 0.00044 | 4.758194 | 0.0047 | 3.519591 | 0.0218 | 0.846199 | 0 | 0.710828 | 0 |
| 9066 | 7.13E-05 | 19.74622 | 0.002235 | 0.000143 | 21.49752 | 0.000317 | 2.482854 | 0.1587 | 2.905271 | 0.073 | 0.86191 | 0 | 0.65628 | 0 |
